# Factors of crisis and culture in international and Chinese death education research: a comparative bibliometric analysis

**DOI:** 10.3389/fmed.2026.1818464

**Published:** 2026-05-22

**Authors:** Yufei Lin, Yan Liu, Jianting Cao, Ziping Pang, Xinran Song, Fangping Dang

**Affiliations:** School of Nursing, Lanzhou University, Lanzhou, Gansu, China

**Keywords:** bibliometrics, citespace, death education, life and death education, visual analysis

## Abstract

**Introduction:**

The sensitive nature of mortality has hindered the death education. However, post-pandemic existential anxiety has drawn significant attention, offering a critical opportunity for disciplinary advancement. Despite this momentum, the field's interdisciplinary nature lacks a unified theoretical framework, urgently necessitating clear development directions. Furthermore, given the profound cultural divergences between Chinese and Western death education paradigms, analyzing these two contexts both independently and comparatively is essential to comprehensively guide the field's future development.

**Design:**

Comparative bibliometric analysis.

**Participants:**

2,311 publications were included.

**Objective:**

This study aims to analyze and compare the evolutionary trajectories, thematic distributions, and emerging frontiers of death education research in international and Chinese contexts. By synthesizing their characteristic data, it delineates the developmental trends in the post-pandemic era and the cultural divergences between Eastern and Western paradigms, thereby providing reference and guidance for the field's future directions from a bibliometric perspective.

**Methods:**

Literature was retrieved from international databases (Web of Science, PubMed, Scopus; timspan: 1971–2025), and a Chinese database (China National Knowledge Infrastructure; timespan: 1980–2025). Utilizing CiteSpace with one-year time slices, we performed co-occurrence, cluster, timeline, burst detection analyses across each bibliometric data fields. Node selection parameters were individually configured based on data density to ensure optimal network visualization. Finally, the meaningful resulting characteristic data were compared and synthesized.

**Results:**

Analysis of 2,311 publications identifies 2020 as a pivotal turning point for significant growth in both international and Chinese death education research. Post-2023, international publication volume continues to accelerate, whereas Chinese research exhibits a downtown trend. Bibliometric results show that the highest betweenness centrality in international research remains anchored in early foundation literature. In contrast, Chinese research displays a marked preference regarding research subjects and reveals that “life education” has superseded “death education” as the primary thematic cluster.

**Conclusion:**

Following a post-2020 surge, international research has maintained sustained growth, whereas Chinese publications experienced a decline post-2023. Distinctively, Chinese studies show a preference for convenience sampling, while international co-citation networks reveal a persistent reliance on early foundational research. To ensure sustainable development, the field must refine theoretical frameworks globally. Furthermore, reversing the decline in China requires leveraging culturally positive semantics and policy directives to position death education as a systemic public health intervention.

**Social media abstract:**

Research intensity of death education in both regions exhibited high synchrony with major public emergencies. International research showed sustained post-pandemic growth but a paucity of theoretical innovation, while Chinese research faced significant localization bottlenecks, characterized by reliance on convenience sampling and a tendency to reframe death discourse through the indirect lens of “life education.”

## 1 Introduction

While the interdisciplinary nature of death education has resulted in a lack of a universally standardized definition, this study references the classic framework proposed by Wass et al. (1) in 1980. Wass mentioned that death education can be defined as the science of formal teaching or instruction of a group or groups about topics related to the subject of “death” including goals and objectives, curriculum content, materials and organization, teaching methods and techniques, and evaluation of teaching outcomes or effectiveness. A broader definition of death education (or any kind of education) would include consideration of the group to be taught, that is, it would consider certain special conditions, characteristics, and needs of the members of the group that may be relevant to the goals and purposes, the subject matter, and the methodology used (1). Therefore, this study operationally defines death education in this work as a multidimensional educational process aimed at enhancing individuals' awareness of death and their coping mechanisms. It is demonstrated that death education is critically important for personal development, professional training, and psychological wellbeing across various populations (2). It can effectively improve emotional recognition and communication, strengthens psychological anchors to navigate existential anxieties (3–5), thus supporting society wellbeing.

Drawing upon the integrated analytical framework proposed by the renowned scholar Testoni, this study contextualizes the role of death education within the current global landscape. In recent years, major global events such as the COVID-19 pandemic have triggered widespread existential anxiety. According to Terror Management Theory (TMT), the constant conflict between survival instinct and the realization of inevitable mortality (called mortality salience) generates intense cognitive dissonance and psychological suffering, prompting individuals to continuously attempt to reduce mortality salience itself (6). From the TMT point of view, therefore, mortality salience caused by the pandemic plays a central role in driving the attitudes and behaviors of most of the population in each country plagued by the virus (7–10). This impact aligns with observations from *the Lancet*: “A pandemic is a cause and powerful amplifier of suffering, through physical illness and death, through stress and anxieties, and through financial and social instability. Alleviation of that suffering, in all its forms, needs to be a key part of the response (11)”. Against this backdrop, death education has garnered increasing attention as a viable response strategy.

However, as a multidisciplinary field spanning psychology, medicine, and sociology, death education lacks a unified theoretical framework (12–14) and a consensus on its developmental trajectory. Given the inherent research challenges stemming from sensitive nature of mortality, it is imperative to capitalize on the current surge in attention to promptly clarify the field's future directions. Therefore, this study aims to conduct a precise quantitative analysis of its evolutionary pathways, underlying drivers, and critical research lacunae, providing a reference for its future development from a bibliometric perspective.

Bibliometrics, a statistical methodology for quantitatively assessing scholarly literature (15), offers a vital complement to qualitative evaluations. Its core value lies in its capacity to identify relational strengths among data points, evolutionary trends, and the velocity of conceptual shifts. By doing so, it provides a crucial quantitative perspective to reference and guide the discipline's future developmental trajectory.

Visualization-based bibliometric studies on death education are still limited. Existing research mainly focuses on describing the current status (16, 17), identifying research hotspots (18, 19), analyzing evolutionary trends (20), andexploring the differences between medical and non-medical research (21). Importantly, most current bibliometric papers tend to simply describe individual analysis results, lacking an overall summary and an indepth discussion of their practical meanings. It is worth noting that there is controversy regarding the trend of research interest in this field since 2020 (21, 22).

Therefore, we intend to conduct a bibliometric analysis of death education research from database inception to the present. Rather than simply describing individual results, our focus is on synthesizing these findings to extract valuable disciplinary characteristics and to provide a new reference for the aforementioned controversy. Furthermore, given the profound cultural divergences between Chinese and Western death education paradigms, analyzing these two contexts both independently and comparatively is essential to comprehensively guide the field's future development. Therefore, this study pioneers a comparative bibliometric analysis designed to systematically map and contrast the knowledge landscape of death education research in international and Chinese contexts. Specifically, the main objectives of this study are: (1) to compare the historical development trajectories, research theme distributions, and emerging frontiers of death education between the international and Chinese contexts; (2) to clarify the trends in research interest post-2020 and analyze the underlying reasons for the aforementioned controversial findings; and (3) to identify and interpret the developmental characteristics and differences across these two cultural backgrounds, thereby providing an objective reference and guidance for future research directions. The scope of this analysis is delimited to literature sourced from major international databases (Web of Science, PubMed, Scopus) and a Chinese database (China National Knowledge Infrastructure), spanning from database inception to Octorber 1, 2025.

## 2 Methods

As this is a bibliometric study based on published scientific evidence extracted from publicly available databases, ethics committee approval was not required. Furthermore, all data collection and analysis strictly adhered to the terms of use and copyright guidelines of the respective databases, ensuring the ethical utilization of data.

### 2.1 Literature search and selection

#### 2.1.1 Data retrieval

To ensure comprehensive coverage and enable cross-cultural comparison, we systematically retrieved publications from four major databases: the Web of Science Core Collection, PubMed, Scopus and the China National Knowledge Infrastructure. The time span was set from the inception of each database to October 01, 2025.

We employed a bilingual search strategy to capture the global literature in English and the distinct body of work in Chinese, as preliminary searches indicated that Chinese publications constitute a significant proportion of the global literature on death education, the exclusion of which would result in selection bias. The reproducible search strings for each database is provided in Supplementary Table A.1.

#### 2.1.2 Data screening

The initial search yielded 4,136 records. After removing ineligible document types (meeting abstracts, editorials, letters, book chapters, news, and retracted papers), 3,839 records remained. These records were imported into EndNote 21 for centralized management. Duplicate removal was performed in two stages: first, using EndNote's built-in “Find Duplicates” function, followed by a meticulous manual inspection to ensure accuracy, resulting in 2,920 unique records.

To minimize subjectivity during literature screening, we established a strict operational definition: death education is operationally defined as any explicitly described educational intervention, curriculum, or training program encompassing comprehensive pedagogical elements, such as specific objectives, tailored methodologies, and outcome evaluations, aimed at enhancing individuals' awareness of death and improving their coping mechanisms. Conversly, studies were excluded according to the following criteria: Content mismatch: studies not primarily focused on death education (e.g., those focusing on Alzheimer's pathology/Tau protein, clinical mortality trends, or stroke thrombolysis); Target setting mismatch: studies not centered on human nursing or medical education contexts (e.g., those mainly involving veterinary medicine, zoo animal behaviors, or natural language processing models); Biographical focus: studies centering on personal histories or tributes (e.g., tributes to Hannelore Wass). To ensure the reliability of the screening process, two researchers independently reviewed the titles, abstracts, and full texts of the retrieved articles against the predefined inclusion and exclusion criteria, ensuring complete reviewer independence. Any discrepancies or disagreements during the screening process were initially addressed through discussion between the two reviewers. If consensus could not be reached, a third senior researcher was consulted to arbitrate and make the final decision.

Finally, the data of 2,311 studies were exported. To ensure compatibility, records from PubMed and Scopus were converted into format of the Web of Science using CiteSpace's built-in converter to create a unified dataset for the subsequent analysis. Given the significant linguistic and formatting differences, the bibliometric analysis was conducted in two separate streams: the English dataset (the Web of Science Core Collection, Scopus, and PubMed combined) and the Chinese dataset (China National Knowledge Infrastructure). It should be noted that citation-based analyses (e.g., co-citation networks analyses) were not performed on the PubMed and China National Knowledge Infrastructure data because data. This is because the standard data export formats of these specific databases do not natively provide the complete cited reference lists required for constructing citation networks. To address this limitation and ensure comparability, the cross-dataset comparison in this study is based strictly on semantic metadata (i.e., keywords, titles, and publication years), which are consistently indexed across all four databases. Consequently, core analyses such as keyword co-occurrence and clustering remain unaffected by reference export discrepancies. Furthermore, retaining both PubMed and the China National Knowledge Infrastructure is methodologically essential to prevent severe selection biases. Specifically, PubMed is an indispensable repository for biomedical literature, omitting it would exclude critical clinical perspectives that are central to death education. Similarly, as Chinese publications constitute a substantial proportion of the literature on death education, excluding them would result in significant geographical and cultural bias. This design thus allows for holistic and reliable analyses while bypassing data incompatibility.

#### 2.1.3 Rationale for data source selection

This study selected the Web of Science Core Collection, PubMed, Scopus, and China National Knowledge Infrastructure as data sources. We employed a multi-database strategy to ensure comprehensive coverage of the literature based on the following considerations. First, the Web of Science Core Collection was selected as the primary data source, given its recognition as the gold standard for high-quality bibliometric analysis and its comprehensive coverage of the most influential academic literature in the field. Second, PubMed was included because it serves as the premier global repository for biomedical and life sciences literature, ensuring the exhaustive coverage of nursing and clinical education research of this study. Third, Scopus was used to complement the other databases by offering the most extensive coverage of peer-reviewed literature, particularly ensuring the inclusion of nursing journals and social science perspectives not indexed in the Web of Science Core Collection and PubMed. Forth, China National Knowledge Infrastructure was recognized as the most authoritative and comprehensive academic repository in China, was incorporated to mitigate language bias and capture the unique cultural nuances of death education in the Chinese context, offering distinct Eastern pedagogical insights that are often underrepresented in English-language databases.

### 2.2 Data standardization and cleaning

To avoid data fragmentation, we performed a rigorous data cleaning process by using CiteSpace's alias function according to the following rules. For country or region names: (i) Abbreviation standardization: variations were standardized to internationally recognized names (e.g., “United States” was unified as “USA”); (ii) Regional merging: regions were harmonized with their sovereign states (e.g., “England”, “Scotland”, “Wales”, “North Ireland” and “London” were reclassified as “UK”). For keywords: (i) Semantic merging: synonyms representing the same concept were combined (e.g., “attitude toward death” and “attitudes death” were merged into “attitude to death”); (ii) Morphological unification: singular forms were converted to plural forms (e.g., “nursing student” to “nursing students”). For institutions: (i) Synonym consolidation: inconsistent variants referring to the same institution were merged into a single standardized primary name (e.g., “Chinese University of Hong Kong” was unified as “Chinese Univ Hong Kong”). The detailed merge lists for countries, keywords and institutions are provided in Supplementary Table A.2, Supplementary Table A.3, and Supplementary Table A.4. Additionally, for the international author co-citation analysis, records labeled as “unknown” were excluded to ensure the integrity and accuracy of the network structure. To ensure comprehensive coverage, we initially retained all Keywords Plus Terms (e.g., “education & educational research”). However, prior to the final analysis, a rigorous screening and unification process was implemented to filter generic categories and merge synonyms, thereby ensuring that the results are both scientifically robust and accurately reflective of the field's reality.

### 2.3 Selection and application strategies for visualization tools

To map the intellectual landscape and illuminate the research hotspots and frontier trends of the field, we selected CiteSpace (Advanced version 6.4.R1) as the primary bibliometric tool for network construction and visualization analysis. Citespace was chosen for its distinct advantage in detecting research frontiers, identifying pivotal turning points, and visualizing the evolution of a field over time.

Firstly, Microsoft Excel (Office 2021) was used to manage and standardize the raw data downloaded from the databases, and to plot the annual publication volume line chart to intuitively display the growth trend of the field. Subsequently, IBM SPSS Statistics (version 31.0) was employed to conduct polynomial regression analysis to rigorously model this temporal trend. Initially, exponential growth models were evaluated, but they yielded suboptimal fits. Accounting for the potential presence of non-monotonic fluctuations in the publication trend, a polynomial regression approach was ultimately adopted. Polynomial regression is appropriated for datasets where the independently variable represents an observation index, such as time (23). Using a polynomial of a lower degree is advantageous as it produces a smoother curve and avoids the severe overfitting risks associated with high-degree polynomials (23). Thus, a third-order polynomial was restricted to avoid the overfitting and tail-end extrapolation risks associated with higher-degree models. The overall feasibility and statistical necessity of this chosen model were evaluated using the coefficient of determination (*R*^2^) and the significance level of the highest-order term.

Secondly, we leveraged CiteSpace's comprehensive capabilities to construct and visualize networks across multiple dimensions, including co-occurrence analysis (for countries, institutions, authors, and keywords) and co-citation analysis (for references, authors, and journals). Specifically, we employed CiteSpace to generate network maps to display structural relationships, cluster maps to reveal thematic concentrations, and timeline views to trace the historical trajectory of the field. Additionally, burstiness detection was conducted on keywords and references to identify emerging trends and abrupt changes. The timespan was set from 1971 to 2025 for the international databases (Web of Science, PubMed, and Scopus), and from 1980 to 2025 for the Chinese database (China National Knowledge Infrastructure), with the time slice consistently set to one year. To ensure the readability and optimal structure of the networks (e.g., avoiding visual clutter caused by excessive links, and preventing isolated nodes due to insufficient connections), the node selection criteria were adjusted and configured differently based on the data density of each analysis. The detailed operational parameters, including the specific selection criteria and pruning, are provided in the respective figure legends. In these visualizations, the size of a node represents frequency or activity intensity, while the thickness of links indicates connection strength. The structural properties of these networks were evaluated using Modularity (*Q*) and Silhouette (*S*) values, where *Q* > 0.3 and *S* > 0.7 signify a significant clustering structure and high cluster homophily, respectively. Furthermore, purple rings highlight nodes with high betweenness centrality (≥ 0.1), indicating potential pivotal points (24) or bridge-spanning works in the network.

**Figure 1:**
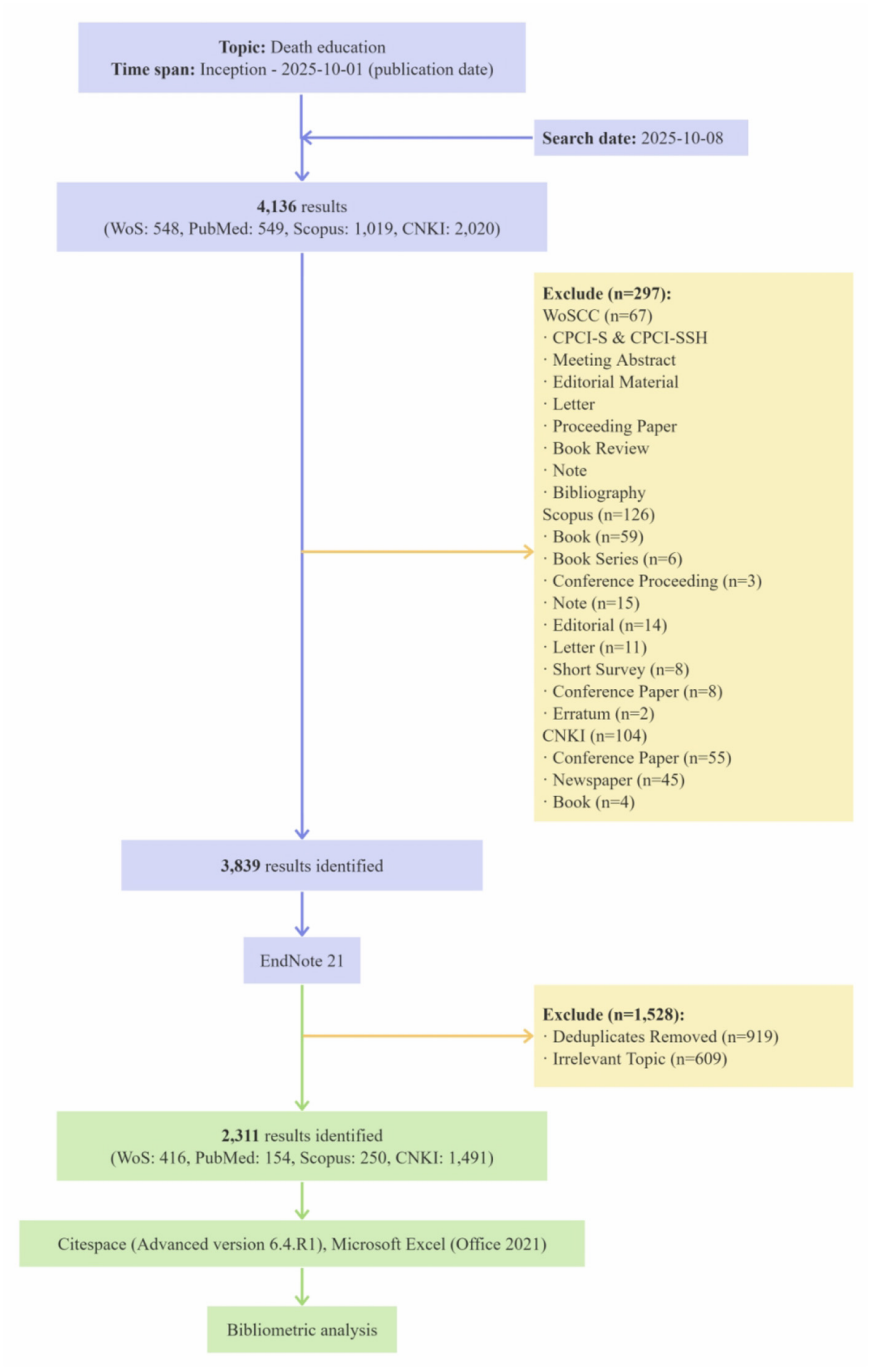
Flowchart for publication identification and analysis.

Although all data fields were analyzed, some fields lacked sufficient node connections, resulting in unclustered, scattered networks which rendered visual analysis meaningless. Therefore, after excluding these fields, we specifically further discussed the visualization results for the two major fields: keyword and author co-citation. The flowchart detailing the paper screening and analysis process is depicted in Figure 1.

## 3 Results

### 3.1 Publication trends

**Figure 2:**
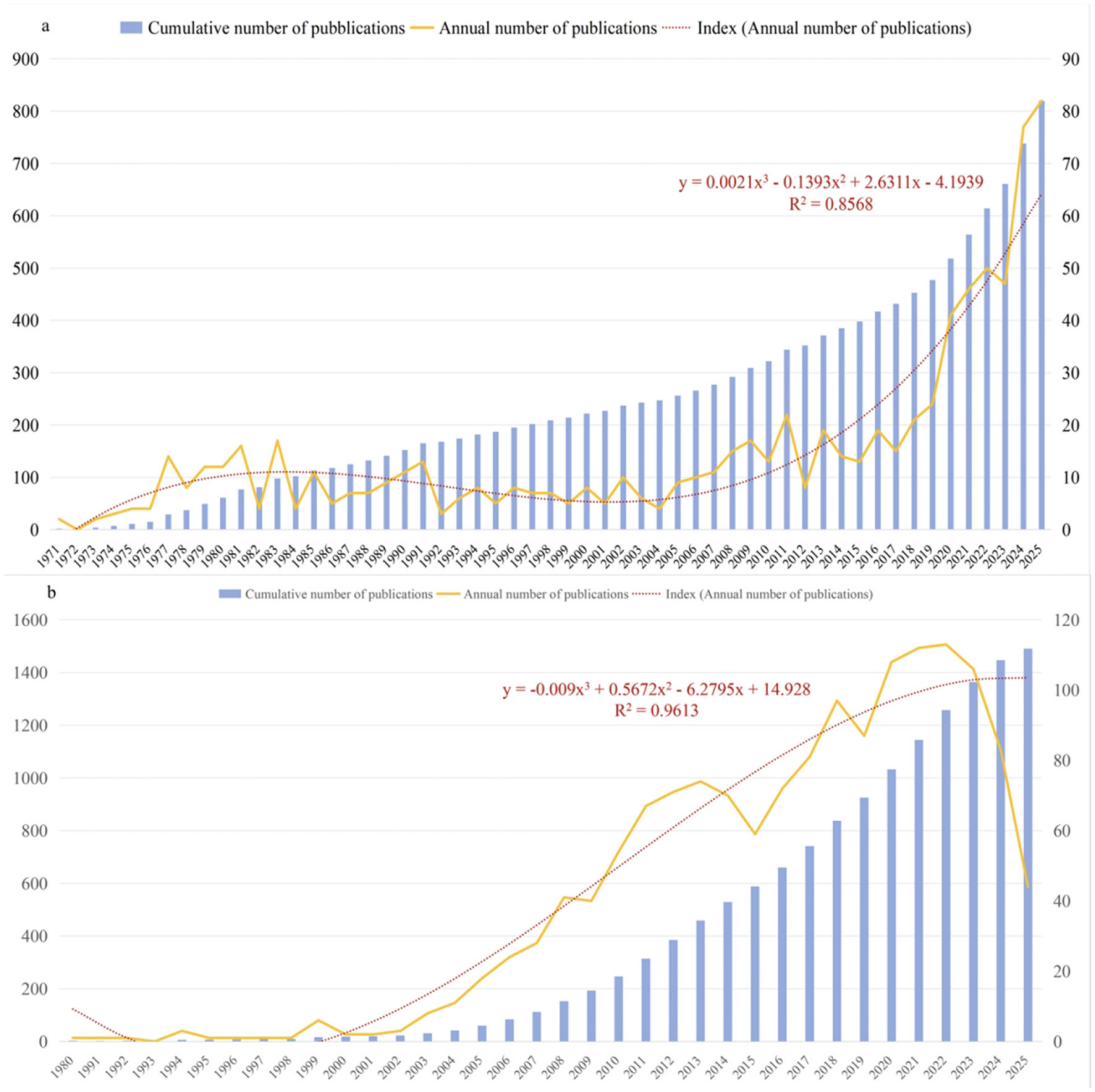
Trends in the number of publications **(a)** Trends in the number of international publications **(b)** Trends in the number of Chinese publications.

The final selection of 2,311 publications contained 820 international publications (35.48%) and 1,491 Chinese publications (64.52%). Figure 2 illustrates the trends of international and Chinese publications on death education from inception to 2025-10-01 (publication date). It can be observed that before 2020, the annual number of international publication experience minor fluctuations below 25; however, in 2020, it exceeded 40 publications for the first time and has continued to grow, surpassing 80 publications in 2025. The average annual publications rose from 18.4 ± 3.98 (2015-2019) to 52.2±12.73 (2020-2024), with an independent samples *t*-test indicating a significant difference [*t* = 5.067, *P* < 0.001, Cohen's *d* = 3.205, 95% *Cl* (18.42, 49.18)]. In contrast, the annual publication curve for China has exhibited substantial, cyclical trends of growth and decline since its early stages. Notably, the most recent upward turning point also occurred in 2020, which then shifted into a downward trend beginning in 2023. We employed a third-order polynomial to fit the publication volume up to 2024 and extrapolated one year forward to predict the 2025 value, yielding a satisfactory goodness of fit (*R*^2^= 0.8568 and 0.9613, respectively). These results indicate that the mathematical model accounts for over 85.68% and 96.13% of the variance in publication volume, demonstrating that the selected models provide a good fit for the historical publication trends. Furthermore, the cubic term coefficients were found to be highly significant (*p* < 0.001), which confirms the necessity of adopting this specific polynomial degree over linear or quadratic alternatives. Specifically, the cubic coefficient in the trend line for international publications is positive (0.0021), resulting in an upward curvature that signifies a phase of accelerated growth. Conversely, the cubic coefficient for China is negative (−0.009), leading to a downward curvature at the tail end of the curve, which implies a weakening of growth momentum or the onset of a decline phase. Global literature on death education dates to 1971 and has since demonstrated sustained research output. In contrast, research in China commenced later, beginning in 1980. Between 1980 and 2000, there were seven years where the annual publication output was only 1. Beginning around 2003, China's annual publication volume started to grow substantially, and in 2012, its cumulative publication volume surpassed that of the international field for the first time, maintaining this lead to the present.

### 3.2 Analysis of keywords

Keywords reveal the core theme of the research. By employing visualization techniques, we can effectively identify evolutionary patterns, prominent research directions and emerging frontiers within the domain.

#### 3.2.1 Co-occurrence analysis of keywords

**Figure 3:**
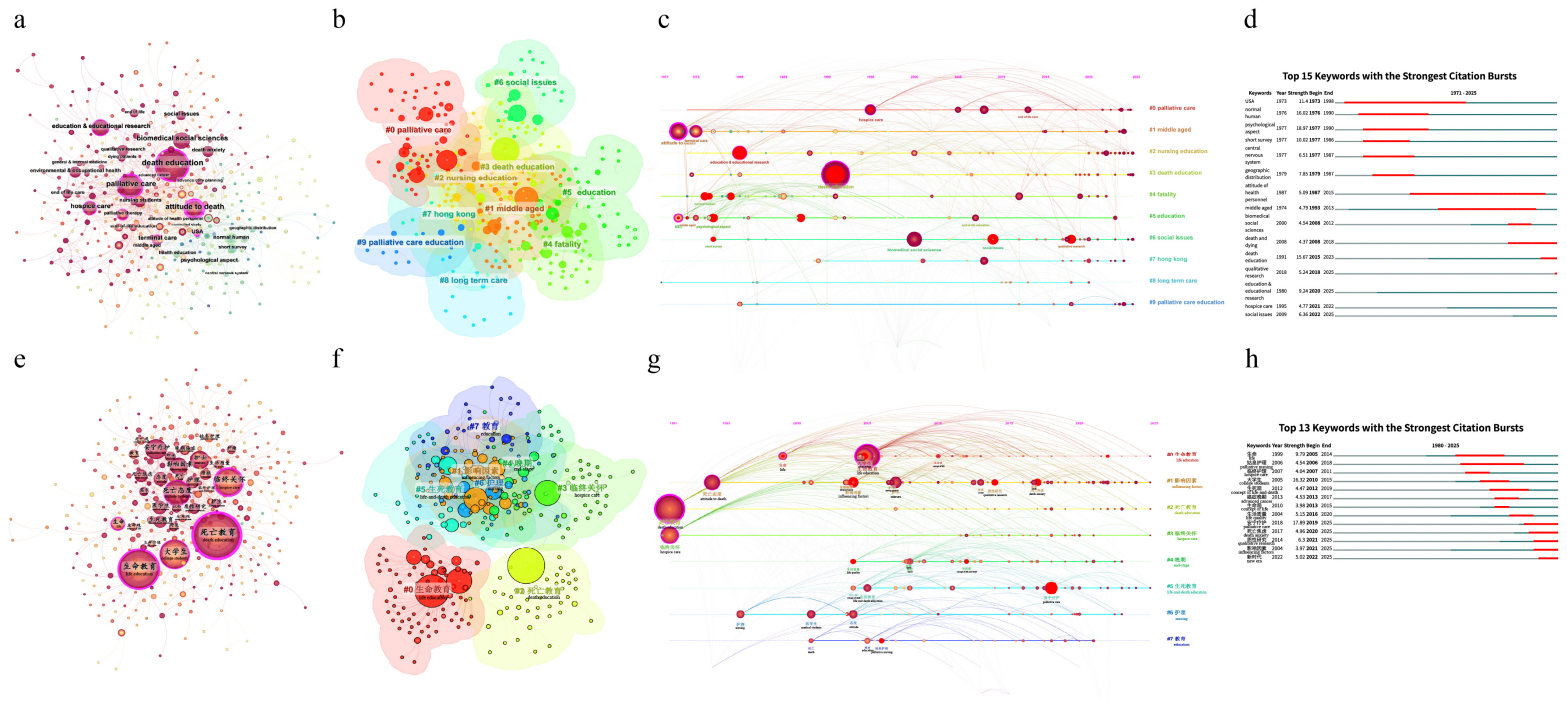
Visualization of keywords **(a)** International co-occurrence analysis **(b)** Chinese co-occurrence analysis **(c)** International clustering analysis **(d)** Chinese clustering analysis **(e)** International timeline analysis **(f)** Chinese timeline analysis **(g)** International burst detection analysis **(h)** Chinese burst detection analysis. Operating parameters for international networks: timespan: 1971–2025 (slice length = 1), selective criteria = g-index (k = 12, LRF = 2.5, L/N = 10, LBY = 5, e = 1.0), pruning = pathfinder. Operating parameters for Chinese networks: timespan: 1980–2025 (slice length = 1), selective criteria = g-index (k = 15, LRF = 2.5, L/N = 10, LBY = 5, e = 1.0), pruning = pathfinder.

To identify research hotspots in the field of death education, we performed keyword co-occurrence analysis (Figures 3a, b), with the top 10 keywords summarized in Table 1. Analyses reveal that in both the international and Chinese research networks, “death education” emerges as the largest node (*n* = 236 and 402). In the figures, the size of each node represents the frequency of the keywords, and the purple rings highlight nodes with high betweenness centrality (> 0.1), indicating potential pivotal points or bridge-spanning works in the network. The link thickness reflects the strength of association between themes, and the temporal color gradient of the nodes illustrates the chronological evolution of research.

**Table 1:** Top 10 most frequent keywords in the international and Chinese field.

Rank	Keywords	Frequency	Centrality	Year
International				
1	Death education	236	0.27	1991
2	Attitude to death	173	0.59	1973
3	Palliative care	128	0.23	1995
4	Biomedical social sciences	94	0.07	2000
5	Hospice care	65	0.18	1995
6	Education & educational research	63	0.10	1980
7	Terminal care	53	0.13	1975
8	Usa	46	0.26	1973
9	Social issues	38	0.06	2009
10	Psychological aspect	37	0.05	1977
Chinese				
1	Death education	402	0.49	1991
2	Life education	327	0.29	2005
3	Hospice care	187	0.38	1991
4	College students	184	0.15	2005
5	Attitude to death	158	0.18	1994
6	Palliative care	86	0.07	2018
7	Influencing factors	86	0.03	2004
8	Nurses	76	0.05	2007
9	Nursing	65	0.08	1996
10	Life	60	0.12	1999

In international publications, “death education” (236), “attitude to death” (173), and “palliative care” (128) emerged as the most frequent keywords, with “USA” being the only country among the high-frequency terms. Notably, although “attitude to death” ranked second in frequency, it yielded the highest betweenness centrality (0.59), far exceeding that of the most frequent keyword “death education” (0.27), indicating its role as a potential pivotal point or bridging concept within the network. In contrast, within Chinese publications, the most frequent nodes were “death education,” “life education” (327) and “hospice care” (187). “Death education” also exhibited the highest betweenness centrality (0.49). In international publications, the top three most frequent study populations were “normal human” (33), “nursing students” (24), and “middle aged” (18). Conversely, in Chinese publications, they were “college students” (183), “nurses” (76), and “medical students” (59).

#### 3.2.2 Clustering analysis of keywords

To further categorize the thematic structure and identify core subfields within the death education domain, we performed keyword cluster analysis using the Log-Likelihood Ratio (LLR) algorithm (Figures 3c, d). Detailed characteristics of these clusters are summarized in Supplementary Table A.5.

In the international network, the modularity (*Q*) was 0.5719 and the weighted mean silhouette (*S*) was 0.8384. Both indices exceeded the respective thresholds of 0.3 and 0.7, indicating a statistically significant clustering structure and a high degree of internal consistency among the nodes within each cluster. Similarly, the Chinese network yielded a modularity (*Q*) of 0.5232 and a silhouette (*S*) of 0.7818. Although these values were slightly lower than those of the international network, they likewise confirmed the high reliability and structural robustness of the clustering results. In the international network, the largest clusters were identified as “#0 palliative” (65 nodes), “#1 middle aged” (59 nodes), and “#2 nursing education” (49 nodes), indicating the main areas of research focus. In particular, “#7 Hong Kong” emerged as an independent keyword cluster, indicating a distinct grouping of keywords related to this specific geographic region. Regarding the Chinese network, the largest clusters were “#0 life education” (70 nodes), “#1influencing factors” (50 nodes), and “#2 death education” (47 nodes). The time span of all the international clusters extends across nearly four decades, from 1982 to 2019. In contrast, the temporal coverage of all the Chinese clusters is densely concentrated between 2010 and 2015, reflecting a dense formation of thematic groupings during these years.

#### 3.2.3 Timeline analysis of keywords

To characterize the evolutionary trends of these themes over time, we generated a keyword timeline visualization (Figures 3e, f). In the figures, nodes highlighted in red denote keywords with high burst strength.

At the inception of the international timeline, large nodes were predominantly situated within Clusters #1 (middle aged) and #5 (education). As early and high-frequency nodes in the timeline, “attitude to death,” “terminal care,” and “USA” reflect the initial thematic foci and the occurrence of keywords related to specific geographic regions. In the post-2000 period, the newly emerged high-frequency nodes are predominantly concentrated in Cluster #6 (social issues), specifically “biomedical social sciences” “qualitative research,” indicating concentrated research centered around new paradigms and methods. In the Chinese timeline, the earliest large nodes were predominantly situated within Clusters #2 (death education) and #3 (hospice care). As early and high-frequency nodes, “death education” and “hospice care” reflect the foundational thematic foci in the Chinese context. Between 2000 and 2010, the newly emerged major nodes, such as “life education,” “college students,” and “nurses,” are predominantly concentrated in Cluster #0 (life education). In the post-2010 period, large nodes are predominantly concentrated within Clusters #1 (influencing factors) and #5 (life-and-death education), specifically represented by terms such as “qualitative research,” “attitude to death,” and “palliative care,” reflecting a concentration of studies focused on these specific methods and themes within this timeframe.

#### 3.2.4 Burst detection analysis of keywords

To detect research frontiers and emerging trends in this field, we conducted keyword burst detection analysis (Figures 3g, h). In the figures, red segments indicate the duration of significant citation bursts, while blue segments represent the time internals of keyword occurrences. The temporal migration of these red segments reflects the evolution and succession of research hotspots over time.

As of 2025, keywords with ongoing citation bursts in the international literature include “qualitative research,” “education & educational research,” and “social issues”; meanwhile, “palliative care,” “death anxiety,” “qualitative research,” “influencing factors,” and “new era” exhibit continuing burstiness in the Chinese literature. These terms indicates the most recent thematic trends and areas of concentrated interest within the field of death education.

### 3.3 Analysis of cited authors

Author co-citation analysis reveals the intellectual base and underlying logic of the field. Visualizing the co-citation network effectively identifies seminal scholars foundational to the discipline's development, as well as their respective research field.

#### 3.3.1 Co-occurrence analysis of cited authors

**Figure 4:**
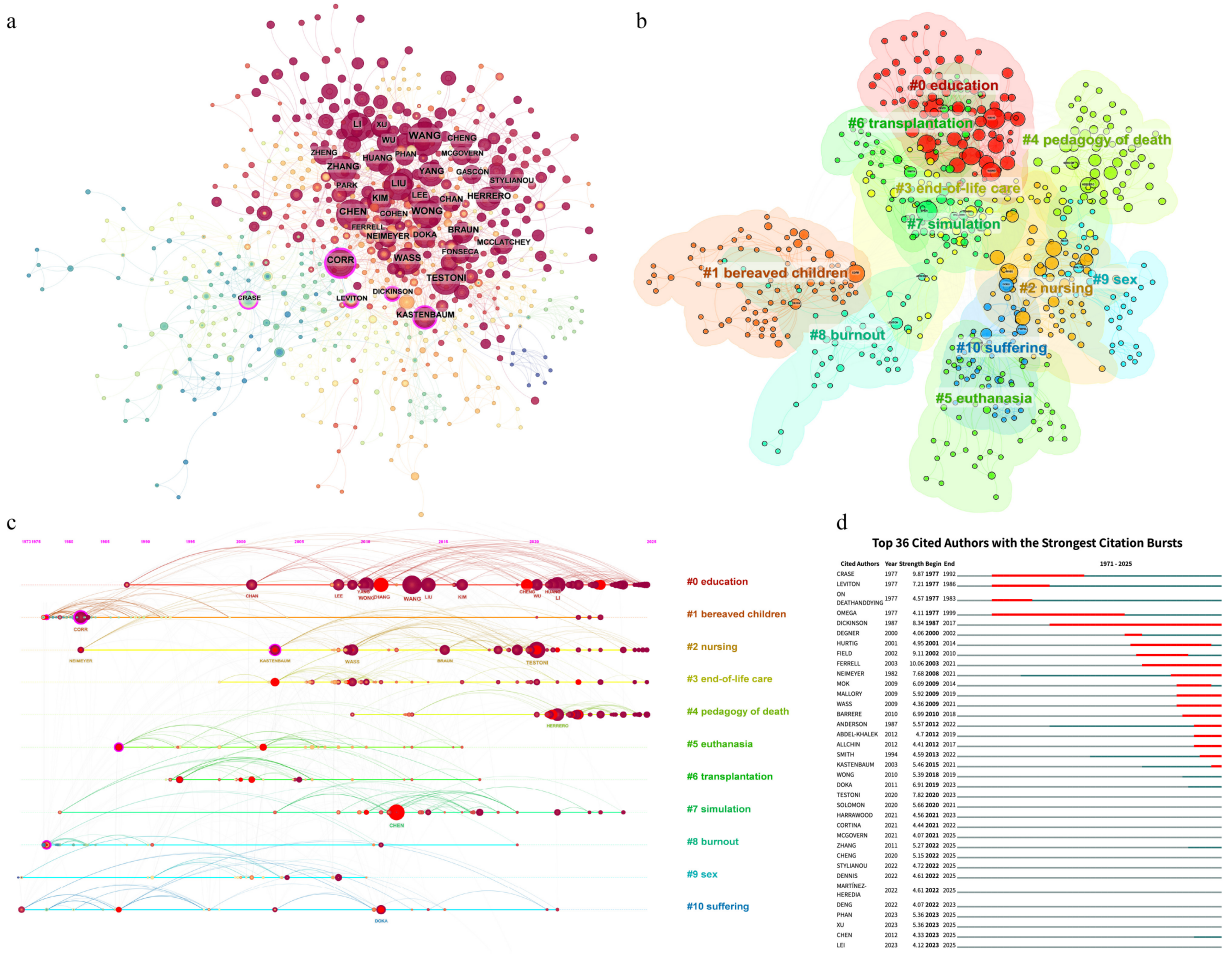
Visualization of cited authors **(a)** Co-occurrence analysis **(b)** Clustering analysis **(c)** Timeline analysis **(d)** Burst detection analysis. Operating parameters: timespan: 1971–2025 (slice length = 1), selective criteria = g-index (k = 15, LRF = 2.5, L/N = 10, LBY = 5, e = 1.0), pruning: pathfinder.

To specifically identify these foundational figures, we conducted an author co-citation analysis on the international dataset (Figure 4a).

Detailed metrics of the top 10 most cited authors are presented in Supplementary Table A.6. WANG (91 citations, 2013), CHEN (75, 2012), and WONG (73, 2010) ranked the highest in citation frequency, corresponding primarily to more recent years. In contrast, the highest betweenness centrality was held by CORR (0.21, 1982), DICKINSON (0.18, 1987), and CRASE (0.17, 1977), indicating their roles as potential pivotal points and bridging nodes within the network. This is manifested in the network structure: lighter-colored early nodes (low-left) are linked to darker, recent nodes (upper-right) via central nodes distinguished by purple rings.

#### 3.3.2 Clustering analysis of cited authors

To identify distinct thematic groupings among the cited authors, we performed cluster analysis on the international dataset using the LLR algorithm (Figure 4b). Detailed characteristics of these clusters are summarized in Supplementary Table A.7.

The network yielded a Modularity Q of 0.7359 and a Weighted Mean Silhouette S of 0.8933, indicating a highly statistically significant clustering structure and robust statistical reliability. Cluster #0 (education), #1 (bereaved children), and #2 (nursing) represent the themes focused on by the most highly cited authors. The average citation years of Clusters #0 (education) and #4 (pedagogy of death) are after 2020, indicating recent citation preferences.

#### 3.3.3 Timeline analysis of cited authors

To delineate the temporal succession of highly cited scholars, we generated an author co-citation timeline view (Figure 4c).

The visualization reveals that nodes with high betweenness centrality identified by purple rings, are exclusively distributed within the earlier periods of the network, such as CORR and KASTENBAUM. Furthermore, it reveals that Cluster #0 (education) exhibits the most enduring citation activity, persisting to the present day; meanwhile, Clusters #2 (nursing) and #4 (pedagogy of death) show recent concentrations of high citation activity, featuring authors such as WANG (Cluster #0), TESTONI (Cluster #2), and HERRERO (Cluster #4).

#### 3.3.4 Burst detection analysis of cited authors

To identify scholars with significant recent citation bursts, we conducted an author co-citation burst detection analysis (Figure 4d).

As of 2025, ongoing citation bursts were observed for ten authors: MCGOVERN, ZHANG, CHENG, STYLIANOU, DENNIS, MARTINEZ-HEREDIA, PHAN, XU, CHEN, LEI. These figures represent the latest and most closely followed authors in the field of death education.

## 4 Discussion

### 4.1 Summary of basic information

This study conducted a bibliometric analysis of 2,311 publications on death education from the databases' inception to October 01, 2025. The data indicate that in the post-pandemic era, the volume of international publications has entered a phase of continuous growth, whereas Chinese publications have begun to decline following an initial increase. Visualization analysis of international countries, institutions, and authors revealed insufficient node connections, resulting in scattered, unclustered networks. These findings indicate a lack of collaborative research in the field of international death education. Regarding the output of countries' institutions, and authors, the leading positions held by USA, the University of Padua, and Testoni I. These foundational findings provide the necessary context for a deeper discussion in the following sections.

### 4.2 The year 2020: an inflection point in both internation and Chinese research

The analysis of the publication trends reveals that 2020 was a significant inflection point, witnessing an abrupt surge in both international and Chinese literature. This abrupt increase chronologically overlapped with the global onset of the COVID-19 pandemic. Additionally, the author co-citation analysis reveals that the mean year of the largest cluster is also 2020. Based on this temporal alignment, we infer that the turning point and subsequent surge in publication volumes starting in 2020 are closely associated with the COVID-19 pandemic. W. Wang et al. (21) identified a similar post-2020 turning point, similarly attributing this momentum to the pandemic. However, while their research primarily focuses on contrasting medical and non-medical perspectives, our study provides an in-depth analysis of the two different post-pandemic developmental trajectories: a sustained growth in international research vs. an initial surge followed by a decline after 2023 in the Chinese context. Conversely, a recent bibliometric analysis by A. Ramos-Pla et al. (22) observed a different trajectory, noting a decrease in publications related to pedagogy of death starting in 2020. This discrepancy may be attributed to methodological differences in database selection and search query design. While their study relied on Web of Science database, our research relied on the Web of Science Core Collection, PubMed, and Scopus for international analysis. Furthermore, their search queries were TS=(“PEDAGOGY OF DEATH” OR “DEATH EDUCATION” OR “DIDACTICS OF DEATH”), while ours were TS=(“death education”) OR TS=(“end-of-life education”) OR TS=(“life-and-death education”). Consequently, these different choices in databases and search queries resulted in their analysis being more oriented toward psychology and pedagogy, whereas our study was more aligned with medical and clinical disciplines.

View through the lens of the pandemic, the surge in death education research is likely driven by two main factors. First, at the individual level, empirical evidence indicates that being impacted by the pandemic (e.g., those with close contacts diagnosed with COVID-19) significantly alters an individual's death attitude, effectively decreasing the long-standing habit of death avoidance (25). Second, at the societal level, studies suggest that the widespread psychological distress triggered by the pandemic has gradually positioned death education as a vital public health coping tool (10, 11). As emphasized by *The Lancet*: “A pandemic is a cause and powerful amplifier of suffering, through physical illness and death, through stress and anxieties, and through financial and social instability. Alleviation of that suffering, in all its forms, needs to be a key part of the response” (11). Building upon this public health imperative, I. Testoni et al. (10) assert that “death education indeed provides an important support also in dealing with the current pandemic situation”. This transition is also reflected in our bibliometric data, notably by the post-2020 burst keyword “social issues.”

The publication data indicates that following the pandemic, international research output did not recede but rather demonstrated growth, whereas Chinese publication volumes experienced a significant decline starting in 2023. This suggests that, compared to Chinese research, international death education research has maintained a more sustainable momentum post-2020. This sustained interest may be attributed to three primary factors. First, psychologically, this can be interpreted through Terror Management Theory (TMT) which suggests that mortality salience caused by the pandemic plays a central role in driving the attitudes and behaviors of most of the population in each country plagued by the virus (7,−9). TMT states that the constant conflict between the survival instinct and the awareness that everyone must die sooner or later (called mortality salience) causes intense cognitive dissonance and suffering to people, who therefore constantly attempt to reduce mortality salience itself (6). The pandemic made this mortality salience impossible to hide (10), causing intense cognitive dissonance and anxiety, which in turn drove a massive societal demand for death education. However, it is worth noting that although TMT is a relatively classic theory, its empirical robustness has recently come under intense scrutiny. Two recent meta-analyses have highlighted significant concerns regarding the mortality salience hypothesis. Specifically, Schindler et al. (26) demonstrated that bias-adjustment techniques suggested the presence of publication bias and/or the exploitation of researcher degrees of freedom and arrived at smaller effect size estimates for the hypothesized interaction, in several cases reducing the effect to non-significance (range gcorrected = −0.36 to 0.15) (26). Similarly, Chen et al. (27) revealed that a synthesis of their findings suggested there were non-zero effects underlying some studies of the MS hypothesis, although the effects were highly heterogeneous, most studies were underpowered, and many individual effects might be spurious (27). Furthermore, a recent empirical investigation by Treger et al. (28) reported that despite using standard procedures, they were unable to replicate basic patterns of the dependent variable in the MS conditions (28). Therefore, a cautious attitude should be maintained regarding TMT. Second, structurally, death education in international context has transitioned from a traditional education model into a broader public health tool, aligning with Testoni's assertions (10). Third, technologically, the proliferation of digital media has effectively lowered the threshold for discussing this sensitive topic (29). In summary, future death education research within the international context should sustain its post-2020 momentum and delve deeper, such as by further advancing the transition of death education into a broader public health tool to satisfy the public's sustained post-pandemic needs. Practically, the transition of death education into a public health tool necessitates targeted professional training. Such training must equip professionals, including healthcare providers, educators and mental health practitioners, with the competencies to deliver death education as a population-level intervention. Consequently, future search can develop these training programs and evaluate the effectiveness of such public-facing interventions.

### 4.3 Convenience preference and the post-2023 sharp retreat in Chinese research

As previously discussed, while international publication volumes have continued to grow in the post-pandemic era, Chinese research experienced an initial surge starting in 2020, followed by a significant decline beginning in 2023. This suggests that, compared to the international context, there may be a more persist bottleneck in Chinese death education, which has made it difficult to sustain the research momentum gained after 2020.

Specifically, Chinese death education research exhibits two distinct characteristics that differentiate it from the international context. First, regarding research populations, Chinese studies exhibit a pronounced demographic preference. In the keyword analysis of Chinese research, the frequencies of “college students” (184), “nurses” (76), and “medical students” (59) far exceed those of “terminally ill patients” (14), “the elderly” (6), and “geriatric patients” (4). Meanwhile, in international research, “middle-aged” has emerged as the second-largest keyword cluster (Cluster #1), with a mean year of 1993. These findings suggest that Chinese research exhibits a preference for utilizing convenience samples, resulting in a concentrated focus on college students and healthcare professionals. Second, regarding conceptual definitions, a significant portion of Chinese research conducts death-education-related studies under the definitions of “life education.” In the keyword analysis, “life education” (327) emerges as the second most frequent node following “death education” (402), and constitutes the largest independent cluster. Furthermore, in the timeline analysis, while early-stage cluster #2 was explicitly labeled “death education,” it was succeeded after 2000 by cluster #0 (life education), which exhibits both a larger scale and a more sustained research duration. Collectively, these pragmatic features, which differ from the international context and aim at ensuring study feasibility, suggest that Chinese research may primarily involve a more persistent avoidance and rejection of death education compared to the West.

We speculate that this phenomenon may be attributed to the hindrance of Western-originated narratives of death education within the Chinese context. For instance, concepts such as “individual autonomy” (e.g., advance directives) and “spiritual care” are not easily understood or integrated within the Chinese linguistic and cultural context. Furthermore, mainstream death education theories are not constructed upon Eastern religious or philosophical foundations, such as Buddhism and Confucianism. This disconnection makes the localized implementation of death education in China difficult to be fully understood and accepted. Reviews (30) and empirical studies (31–33) have also noted this issue. This cultural difference is also reflected in the Cultural Map-WVS wave 7 (2017–2022) (34). In this map, China occupies a high position in Secular-Rational values relative to other countries, indicating that Chinese society places less emphasis on religion, traditional family values, and authority. This seemingly contradicts the conclusions of our analysis. However, the item within the highest factor loading used in the map to determine Traditional values vs. Secular-Rational values is “God is very important in respondent's life.” In contrast, the two major traditional religions in China are Buddhism and Taoism. This indicates that even authoritative existing studies sometimes reflect a neglect of Eastern religions and perspectives, which impacts their findings.

However, two certain clues identified in our study may provide references for advancing Chinese death education research. First, the different translations and expressions of concepts related to death education may impact the difficulty of promoting it in China. Our analysis reveals that as a crucial implementation setting for death education, the Chinese translation of palliative care, “Anning Liaohu,” is the keyword with the highest burst strength. Bursting continuously from 2019 to the present, it has garnered rapidly growing scholarly attention. Although this term may have been mentioned in early death education research, it was not until 2018 that it passed the frequency threshold (*g*-index: *k* = 15) to emerge as a valid keyword node. While this growth may be partially attributed to a 2017 official practice guideline (35) that unified related Chinese expressions into “Anning Liaohu,” its burst intensity (17.89) far exceeds the historical burst values of its predecessors, such as “Guxi Huli” (4.54) and “Linzhong Huli” (4.04). This suggests that “Anning Liaohu” may posses unique applicability advantages within the Chinese context. This superiority stems from the inherent connotations of “peace” and “comfort” in its Chinese characters. Furthermore, this inference could potentially be reflected in the observation that the burst duration of “Guxi Huli” (where implies a certain degree of soothing, whereas “Linzhong” directly denotes approaching death) lasted eight years longer than that of “Linzhong Huli.” Second, integrating with China's policies may help promote death education research. Our study found that the keyword “new era” exhibited a high intensity burst immediately upon its first appearance in death education research in 2022, which continuous to the present.

### 4.4 The high structural centrality of early scholars in analysis of cited authors

The author co-citation network reveals a notable structural pattern: while contemporary authors appear as high-frequency nodes, they generally exhibit low centrality. Conversely, nodes possessing high betweenness centrality predominantly first appeared in the analysis prior to the year 2000. Specifically, the highest centrality is held by CORR, DICKINSON, and CRASE. Corr made significant contributions to the advancement of pediatric hospice care (36). DICKINSON extensively investigated healthcare professionals' attitudes toward complex end-of-life issues and the integration of palliative education across diverse medical discipline (37). CRASE championed the systematic integration of death education into formal health education curricula (38). Unlike a typical uniform distribution, the author co-citation network exhibits a distinct seperation between early and recent nodes: lighter-colored early nodes (low-left) are linked to darker, recent nodes (upper-right) via central nodes distinguished by purple rings. While this may partly stem from the continuous citation of classic literature or the sustained activity of early scholars, such an atypical network graph, where old and new nodes are distinctly separated yet linked exclusively by the cohort of scholars who began researching death education in its early stages, potentially indicates a phenomenon in the field of death education: the exact same group of scholars consistently provides the research foundation for subsequent studies. This highlights the need for researchers to more actively engage in the exploration and development of new research foundations. For instance, studies within the Chinese context could be directed toward developing indigenous death education theories grounded in Eastern cultural frameworks.

### 4.5 Limitations

This study is subject to several limitations. First, as a quantitative approach, bibliometric analysis relies primarily on metadata (e.g., titles, abstracts, and citations) and thus cannot evaluate the specific clinical efficacy or the theoretical depth of the individual studies identified. Second, although efforts were made to integrate multi-language databases, relevant literature published in languages other than Chinese and English may not have been comprehensive captured. Third, due to the structural asymmetry and varying citation styles between international (e.g., WoSCC) and regional (e.g., CNKI) databases, achieving perfectly equivalent cross-corpus analyses remains an objective challenge. Forth, bibliometric inference has inherent limitations and cannot substitute for direct empirical research in supporting broad sociocultural interpretations. Based on these limitations, future research could be advanced by conducting empirical studies to further explore and gain a deeper understanding of these sociocultural phenomena.

## 5 Conclusion and prospects

In conclusion, our bibliometric analysis reveals that the post-2020 era marks a critical juncture for death education research, characterized by divergent trajectories. While international scholarship demonstrates sustained growth, Chinese publication volumes experienced an initial surge followed by a sharp retreat post-2023. Despite the sustained international momentum, network analysis highlights the high structural centrality of early scholars, suggesting that there may be a persistent reliance on foundational literature and a potential need for paradigmatic innovation. In contrast, Chinese research exhibits distinct characteristics, notably a convenience preference in sampling and the extensive utilization of “life education” as a conceptual framework. We speculate that these pragmatic features may reflect the potential barriers faced by Western-originated narratives within the Chinese context. To advance Chinese death education research and reverse the recent decline, future efforts may benefit from leveraging culturally positive semantics (e.g., Anning Liaohu) and furthering the transition of death education into a broader public health tool to satisfy sustained societal needs.

## Data Availability

Publicly available datasets were analyzed in this study. This data can be found here: The datasets analyzed for this study are derived from public literature databases, including the Web of Science, PubMed, Scopus, and China National Knowledge Infrastructure (CNKI). Specific search strategies and inclusion criteria are detailed in the Methods section of the article. Therefore, no specific repository link or accession number is applicable.
