# Supplementary material for: Factors of crisis and culture in international and Chinese death education research: a comparative bibliometric analysis
Source: Front Med (Lausanne). 2026 May 22;13:1818464. doi: 10.3389/fmed.2026.1818464 (PMC13237690; doi:10.3389/fmed.2026.1818464)
Supplement: Supplementary file 1 [file Table_1.DOCX]

**Appendix A**

Table A.1 Detailed search strategies and retrieval criteria for four databases

| **Database** | **Search query** | **Filters** |
| --- | --- | --- |
| Web of Science | TS=("death education") OR TS=("end-of-life education") OR TS=("life-and-death education") | Time span: inception - 2025-10-01;  excluded types: Conference Proceedings Citation Index - Science (CPCI-S), Conference Proceedings Citation Index - Social Science & Humanities (CPCI-SSH), meeting abstract, editorial material, letter, proceeding paper, book review, note, bibliography |
| PubMed | ("death education"[Title/Abstract]) OR ("end-of-life education"[Title/Abstract]) OR ("life-and-death education"[Title/Abstract]) | Time span: inception - 2025-10-01 |
| Scopus | TITLE-ABS-KEY("death education") OR TITLE-ABS-KEY("end-of-life education") OR TITLE-ABS-KEY("life-and-death education") | Time span: inception - 2025-10-01;  excluded types: book, book series, conference proceeding, note, editorial, letter, short survey, conference paper, erratum |
| China National Knowledge Infrastructure | **SU='生死教育' OR SU='死亡教育' OR SU='临终教育'** | Time span: inception - 2025-10-01;  excluded types: conference paper, newspaper, book |

Table A.2 List of merged countries

| **Rank** | **Primary Term** | **Secondary Term** |
| --- | --- | --- |
| 1 | AUSTRALIA | AUSTRALIARINGGOLD: 1319, SOUTH AUSTRALIA |
| 2 | BRASIL | BRASIL E-MAIL: OLAVOMAURICIO128@GMAILCOM UNIVERSIDADE FEDERAL DE CAMPINA GRANDE UNIVERSIDADE FEDERAL DE CAMPINA GRANDE CUITÉPB BRAZIL OLAVOMAURICIO128@GMAILCOM, BRASILE-MAIL MARIAHELOYSEMONTEIRO@HOTMAILCOM UNIVERSIDADE FEDERAL DE CAMPINA GRANDE UNIVERSIDADE FEDERAL DE CAMPINA GRANDE CUITÉPB BRAZIL MARIAHELOYSEMONTEIRO@HOTMAILCOM, BRASILE-MAIL: CIDAAVELAR2014@GMAILCOM UNIVERSIDADE FEDERAL DE CAMPINA GRANDE UNIVERSIDADE FEDERAL DE CAMPINA GRANDE CUITÉPB BRAZIL CIDAAVELAR2014@GMAILCOM, BRASILE-MAIL: GAGRA@YAHOOCOMBR UNIVERSIDADE FEDERAL DE CAMPINA GRANDE UNIVERSIDADE FEDERAL DE CAMPINA GRANDE CUITÉPB BRAZIL GAGRA@YAHOOCOMBR, BRASILE-MAILKADLAJORCELI@HOTMAILCOM UNIVERSIDADE FEDERAL DE CAMPINA GRANDE UNIVERSIDADE FEDERAL DE CAMPINA GRANDE CUITÉPB BRAZIL KADLAJORCELI@HOTMAILCOM |
| 3 | UK | BRISTOLMARGARETWHITTLE@UWEACUK, ENGLAND, LONDON, NORTH IRELAND, SCOTLAND, UNITED KINGDOM, WALES |
| 4 | USA | CALIFORNIA, CALIFORNIAELECTRONIC ADDRESS: DRIFKIN@UCSDEDU, ILLINOIS, INDIANA, JOHNSON CITY, NEBRASKA, TEXAS, UNITED STATES, UNITED STATES OF AMERICA, UNITED STATES OF AMERICAELECTRONIC ADDRESS: ASTOKMAN@CSUSTANEDU, UNIVERSITY OF UTAH IN SALT LAKE CITY, USA BRYMOORE@UTMBEDU, USAADAMSK@OHSUEDU, USAEVELINADIFRANCO@CASEEDU, USAKBRAUN@HAWAIIEDU, USAMXP42@CASEEDU, USANANDANMO@MWSCEDU, USARANDERSO@MAILMCWEDU, USAROBINSONRL@HALLAMAREDU, USASBLOCK@PARTNERSORG, USASTACYFISCHER@UCHSCEDU, USATLSMITH@UMBCEDU |
| 5 | CANADA | CANADA T6G 2G3, MANITOBA |
| 6 | CHINA | CHINAELECTRONIC ADDRESS: ZENGYANLI@CDUTCMEDUCN, HONG KONG, MACAO, NEW TERRITORIESMHJMAK@CUHKEDUHK, PEOPLES R CHINA, REPUBLIC OF CHINA, TAIWAN |
| 7 | CROATIA | CROATIAIVASORTA@MEDRIHR |
| 8 | GERMANY | FED REP GER |
| 9 | ISRAEL | ISRAEL ADIR@HADASSAHORGIL |
| 10 | SOUTH KOREA | KOREAHYUNCHAE@PLAZASNUACKR, KOREAJOKW@CUACKR, SOUTH KOREAHJLEE@CUACKR |
| 11 | JAPAN | NAGOYA MEMORIAL HOSPITAL, NAGOYA UNIVERSITY GRADUATE SCHOOL OF MEDICINE, SOPHIA UNIVERSITY |
| 12 | RUSSIA | RUSSIAN FEDERATION |
| 13 | TURKIYE | TURKEY |

Table A.3 List of merged keywords

| **Rank** | **Primary Term** | **Secondary Term** |
| --- | --- | --- |
| 1 | physician-patient relations | *physician-patient relations, doctor patient relation |
| 2 | advance directives | advance directives/ethnology, advance directive |
| 3 | advance healthcare directives | advance health care directive |
| 4 | advanced care planning | *advance care planning |
| 5 | allied health personnel | allied health personnel/*education, allied health personnel/*education/psychology, allied health personnel/*psychology |
| 6 | anxiety | anxiety/*prevention & control/psychology, anxiety/prevention & control, anxiety/etiology/*prevention & control |
| 7 | attitude of health personnel | attitude of health personnel/*ethnology, *attitude of health personnel |
| 8 | attitude to death | attitude to death/*ethnology, attitude toward death, attitude towards death, attitudes toward death, attitudes towards death, attitudes death, *attitude to death, death attitudes analysis |
| 9 | breast neoplasm | breast neoplasms/*psychology |
| 10 | cancer patients | cancer patient |
| 11 | chronic disease | chronic disease/nursing |
| 12 | clinical competence | clinical competence/standards, *clinical competence |
| 13 | college students | university students |
| 14 | communication skills | communication skill |
| 15 | covid - 19 | covid 19 |
| 16 | cross-sectional studies | cross-sectional study |
| 17 | cultural diversity | *Cultural diversity |
| 18 | dacum method | dacum technique |
| 19 | death fear | fear of death |
| 20 | evaluation studies | evaluation studies as topic |
| 21 | geriatric nursing | geriatric nursing/*education |
| 22 | health education | health education/*methods, health education/*organization & administration |
| 23 | health knowledge | *health knowledge |
| 24 | health occupations | health occupations/*education, health occupations/*psychology |
| 25 | health personnel | health personnel/*education, health personnel/*education/psychology, health personnel/*psychology, *health personnel |
| 26 | heart failure | *heart failure/diagnosis/therapy |
| 27 | hospice care | hospice care/*organization & administration, *hospice care |
| 28 | Inservice training | Inservice training/*organization & administration, *inservice training |
| 29 | nurses role | nurses role/*psychology |
| 30 | nursing students | nursing student |
| 31 | palliative care | palliative care/methods/*psychology, *palliative care, *palliative care/methods/psychology |
| 32 | patient education | *patient education as topic |
| 33 | psychological tests | *psychological tests |
| 34 | quality of life | *quality of life |
| 35 | simulation training | *simulation training/methods/standards |
| 36 | social support | *social support |
| 37 | social values | *social values |
| 38 | social work | social work/education |
| 39 | surveys and questionnaires | *surveys and questionnaires |
| 40 | terminal care | terminal care/*psychology, terminal care/psychology, *terminal care, *terminal care/ethics/organization & administration/psychology, *terminal care/methods/psychology/standards, *terminal care/psychology |
| 41 | terminal disease | *Terminally ill |
| 42 | death pedagogy | pedagogy of death |

Table A.4 List of merged institution

| **Rank** | **Primary Term** | **Secondary Term** |
| --- | --- | --- |
| 1 | Chinese Univ Hong Kong | Chinese University of Hong Kong |
| 2 | Arizona State Univ | Arizona State University |
| 3 | Univ Utah | The University of Utah |
| 4 | Univ Maryland | University of Maryland |
| 5 | Western Carolina Univ | Western Carolina University |
| 6 | Zonguldak Bulent Ecevit Univ | Zonguldak Bulent Ecevit University |
| 7 | Natl Taiwan Univ Hosp | National Taiwan University Hospital |
| 8 | Harvard Univ | HARVARD UNIV |
| 9 | Kongyang Univ | Kongyang Univ |
| 10 | Univ Hong Kong | The University of Hong Kong |
| 11 | Cent South Univ | Central South University |
| 12 | Univ Padua | Universita degli Studi di Padova |

Table A.5 Detailed characteristics of clusters identified by Log-Likelihood Ratio (LLR)

| **Cluster ID** | **Cluster Name (LLR)** | **Size** | **Silhouette** | **Mean (Year)** |
| --- | --- | --- | --- | --- |
| International | | | | |
| 0 | palliative care | 65 | 0.699 | 2014 |
| 1 | middle aged | 59 | 0.837 | 1993 |
| 2 | nursing education | 49 | 0.853 | 2007 |
| 3 | death education | 42 | 0.795 | 2011 |
| 4 | fatality | 41 | 0.849 | 1982 |
| 5 | education | 37 | 0.862 | 1992 |
| 6 | social issues | 27 | 0.923 | 2014 |
| 7 | hong kong | 17 | 0.908 | 2006 |
| 8 | long term care | 17 | 0.839 | 2005 |
| 9 | palliative care education | 14 | 0.939 | 2019 |
| 10 | public health and palliative care | 8 | 0.972 | 2016 |
| 11 | history | 7 | 0.996 | 1998 |
| 12 | pulmonary rehabilitation | 7 | 0.973 | 1993 |
| 13 | relaxation therapy | 6 | 0.995 | 1986 |
| Chinese | | | | |
| 0 | life education | 70 | 0.815 | 2012 |
| 1 | influencing factors | 50 | 0.722 | 2015 |
| 2 | death education | 47 | 0.798 | 2014 |
| 3 | hospice care | 38 | 0.622 | 2011 |
| 4 | end-stage | 32 | 0.799 | 2013 |
| 5 | life-and-death education | 32 | 0.751 | 2015 |
| 6 | nursing | 28 | 0.886 | 2011 |
| 7 | education | 28 | 0.782 | 2012 |
| 8 | voluntary euthanasia | 13 | 0.862 | 2013 |
| 9 | advanced cancer | 6 | 0.938 | 2012 |
| 10 | grief counseling | 4 | 0.999 | 2023 |
| 11 | ceremony | 3 | 0.997 | 2014 |

Table A.6 Top 10 most cited authors in the field of death education

| **Rank** | **Cited authors** | **Frequency** | **Centrality** | **Year** |
| --- | --- | --- | --- | --- |
| 1 | WANG | 91 | 0.04 | 2013 |
| 2 | CHEN | 75 | 0.04 | 2012 |
| 3 | WONG | 73 | 0.05 | 2010 |
| 4 | LI | 65 | 0.03 | 2021 |
| 5 | TESTONI | 65 | 0.01 | 2020 |
| 6 | CORR | 63 | 0.21 | 1982 |
| 7 | WASS | 58 | 0.08 | 2009 |
| 8 | LIU | 58 | 0.02 | 2014 |
| 9 | ZHANG | 56 | 0.03 | 2011 |
| 10 | KIM | 47 | 0.02 | 2016 |

Table A.7 Detailed characteristics of clusters of cited authors identified by Log-Likelihood Ratio (LLR)

| **Cluster ID** | **Cluster Name (LLR)** | **Size** | **Silhouette** | **Mean (Year)** |
| --- | --- | --- | --- | --- |
| 0 | education | 84 | 0.846 | 2020 |
| 1 | bereaved children | 80 | 0.955 | 1987 |
| 2 | nursing | 61 | 0.889 | 2015 |
| 3 | end-of-life care | 57 | 0.788 | 2011 |
| 4 | pedagogy of death | 54 | 0.831 | 2021 |
| 5 | euthanasia | 51 | 0.949 | 2003 |
| 6 | transplantation | 41 | 0.900 | 2003 |
| 7 | simulation | 40 | 0.823 | 2014 |
| 8 | burnout | 40 | 0.944 | 1984 |
| 9 | sex | 38 | 0.933 | 1995 |
| 10 | suffering | 38 | 0.908 | 1996 |
| 11 | pre-and-post-seminar tests | 28 | 0.944 | 1985 |
| 12 | nutrition & dietetics | 21 | 0.959 | 1992 |
| 13 | terminally ill patient | 18 | 0.927 | 2005 |
| 14 | intensive-care-unit | 13 | 0.936 | 2013 |
| 15 | experience | 10 | 0.922 | 2012 |

Table A.8 Glossary of principle conceptual and methodological terms

| **Term** | **Definition** |
| --- | --- |
| Betweenness centrality | A metric used to identify pivotal nodes that act as bridges connecting different thematic clusters within the network. |
| Bibliometrics | A statistical methodology for quantitatively assessing scholarly literature, offers a vital complement to qualitative evaluations. |
| Burst | A statistical detection of a sudden surge in the frequency of a node (e.g., citation or keyword) over a specific time interval, signaling a research frontier or emerging trend. |

| Death education | A multidimensional educational process aimed at enhancing individuals’ awareness of death and their coping mechanisms. |
| --- | --- |
| *e* | A threshold to include only nodes with a frequency greater than or equal to *e*. |

| *g*-index | A selection threshold used to filter nodes in each time slice, defined as the largest rank g such that the top g nodes have a cumulative quantity of at least *g^2^*. |
| --- | --- |
| LBY | Look back years, a temporal filtering parameter that restricts citation connections to a specified number of retrospective years, filtering out outdated links to focus on recent interactions. |
| Log-likelihood ratio | A statistical algorithm used to extract cluster labels by identifying terms with the highest probability of representing the unique and core theme of a specific cluster. |
| LRF | Link retaining factor, network configuration parameter that specifies the maximum number of links retained per node, used to simplify the visualization by keeping only the most significant connections. |
| L/N | Links per node, limits the maximum ratio of links to nodes to control network density. |

| Modularity (*Q*) | A structural measure used to evaluate the significance and clarity of a network’s division into distinct clusters. |
| --- | --- |

| Mortality salience | A psychological state or experimental induction where an individual’s awareness of the inevitability of their own death is heightened, typically triggering defensive mechanisms to uphold their cultural worldviews and self-esteem. |
| --- | --- |

| Pathfinder | A network scaling algorithm used to simplify complex networks by removing redundant or less significant links while preserving the most essential structural connections between nodes. |
| --- | --- |
| Pruning | The technical process of filtering out non-essential links from a visualization network to reduce noise and enhance the clarity. |
| Silhouette (*S*) | An index used to assess the internal consistency and degree of homophily within a specific research cluster. |

| Terror management theory | A social psychological framework proposing that the existential conflict between the biological drive for self-preservation and the cognitive awareness of the inevitability of death is managed through the adherence to cultural worldviews and the pursuit of self-esteem. |
| --- | --- |
